# Artificial intelligence in spinal fracture imaging: from clinical application to translational promise

**DOI:** 10.3389/fmed.2026.1916223

**Published:** 2026-10-08

**Authors:** Zhimin Li, Cheng Tang, Peibo Feng, Xiaohan Wang, Heng Zhang, Yuanxiang Lan, Ning Wang

**Affiliations:** 1 Department of Spine Surgery, The First Affiliated Hospital of Guilin Medical University, Guilin, China; 2 School of Intelligent Medicine and Biotechnology, Guilin Medical University, Guilin, China; 3 Department of Plastic Surgery, The First Affiliated Hospital of Xiamen University, Xiamen, China

**Keywords:** artificial intelligence, clinical translation, deep learning, imaging diagnosis, spinal fractures

## Abstract

Artificial intelligence (AI) is reshaping medical imaging, and deep learning together with machine learning are now being used to detect, classify, and differentially diagnose spinal fractures. In this Review, we survey the principal AI architectures that currently support spinal imaging, including convolutional neural networks, object detectors, and hybrid models, and we assess their reported performance for osteoporotic, traumatic, and pathological vertebral fractures. Our synthesis indicates that AI can achieve high diagnostic accuracy in controlled, often single-centre retrospective settings, but its performance consistently declines for mild fractures, degenerated spines, and postoperative anatomy. Critically, the majority of published models lack external validation, and many studies suffer from methodological shortcomings such as selective reporting, insufficient sample sizes, and absence of predefined analysis plans—factors that systematically inflate apparent accuracy. Beyond detection, we explore the growing applications of AI in surgical planning, complication prediction, and functional outcome estimation, while emphasising that most evidence remains preliminary. We identify persistent gaps, including underuse of foundation models and public benchmarks, and limited independent evaluation of commercial tools. We conclude by proposing a shift from performance reporting to methodologically rigorous, prospectively designed studies that incorporate bias assessment, standardised reporting, and patient-centred endpoints—steps essential to translate technical promise into tangible patient benefit.

## 1 Introduction

Spinal fractures encompass a heterogeneous group of injuries encountered across orthopaedics, neurosurgery, geriatrics, and oncology, including osteoporotic fragility fractures in older adults, high-energy traumatic injuries, and vertebral destruction related to metastatic disease (1–4). Imaging remains central to diagnosis, but conventional interpretation is heavily dependent on reader experience and is vulnerable to inter-observer variability, workflow pressure, and missed subtle abnormalities (5–7). These limitations are especially relevant in older patients, in whom mild osteoporotic vertebral compression fractures (OVCFs) may be obscured by degenerative change, chronic deformity, or multimorbidity, thereby delaying osteoporosis evaluation and secondary fracture prevention (5, 8–11). Against this clinical background, AI has emerged as a practical strategy for augmenting spinal imaging by automating lesion detection, localization, classification, and quantitative assessment from large imaging datasets (1, 12–15).

Current evidence suggests that AI can support several clinically relevant tasks in spinal fracture imaging. On CT, deep-learning models have shown strong performance for vertebral fracture detection and triage (1, 16, 17). On MRI, AI systems can assist with lesion characterization, including the distinction between acute osteoporotic fractures, metastatic involvement, hemangioma, and infective or inflammatory mimics (18). Hybrid approaches that combine learned image features with conventional classifiers have also improved differential diagnosis between benign compression fractures and spinal metastases (19). Taken together, these studies indicate that AI is most credible when framed as decision support that complements specialist interpretation, rather than as a stand-alone replacement for expert clinical judgment (20).

Despite these advances, translation into routine care remains constrained by several methodological and clinical barriers. Diagnostic performance is influenced by fracture morphology, anatomical level, image quality, hardware-related artifact, and postoperative change (8, 21). For example, even an FDA-approved vertebral fracture algorithm showed reduced sensitivity for mild Genant grade 1 fractures and lower accuracy in severely degenerated or instrumented spines (5). In addition, many published models are trained on limited, single-center datasets with heterogeneous reference standards and non-uniform evaluation metrics, which undermines external validity (1, 22, 23). The limited interpretability of deep-learning outputs further constrains clinician trust. Accordingly, the next phase of research should emphasize robust external validation, multimodal integration, transparent model design, and prospective assessment in real clinical workflows, with patient-centered outcomes as the ultimate benchmark (1, 20). A major gap in the literature is the absence of direct head-to-head comparisons between AI architectures on shared benchmarks, obscuring whether performance gains reflect algorithmic progress or dataset-specific overfitting.

We acknowledge the methodological limitations inherent to a narrative review, including the potential for selection bias in study inclusion. Our study selection, though systematic in search, reflects author judgment and may be subject to such bias. We have therefore emphasised transparent discussion of these limitations throughout the review, and we caution readers against overinterpreting the reported performance figures, while highlighting potential overestimation and missing validations where relevant (Figure 1).

**Figure 1:**
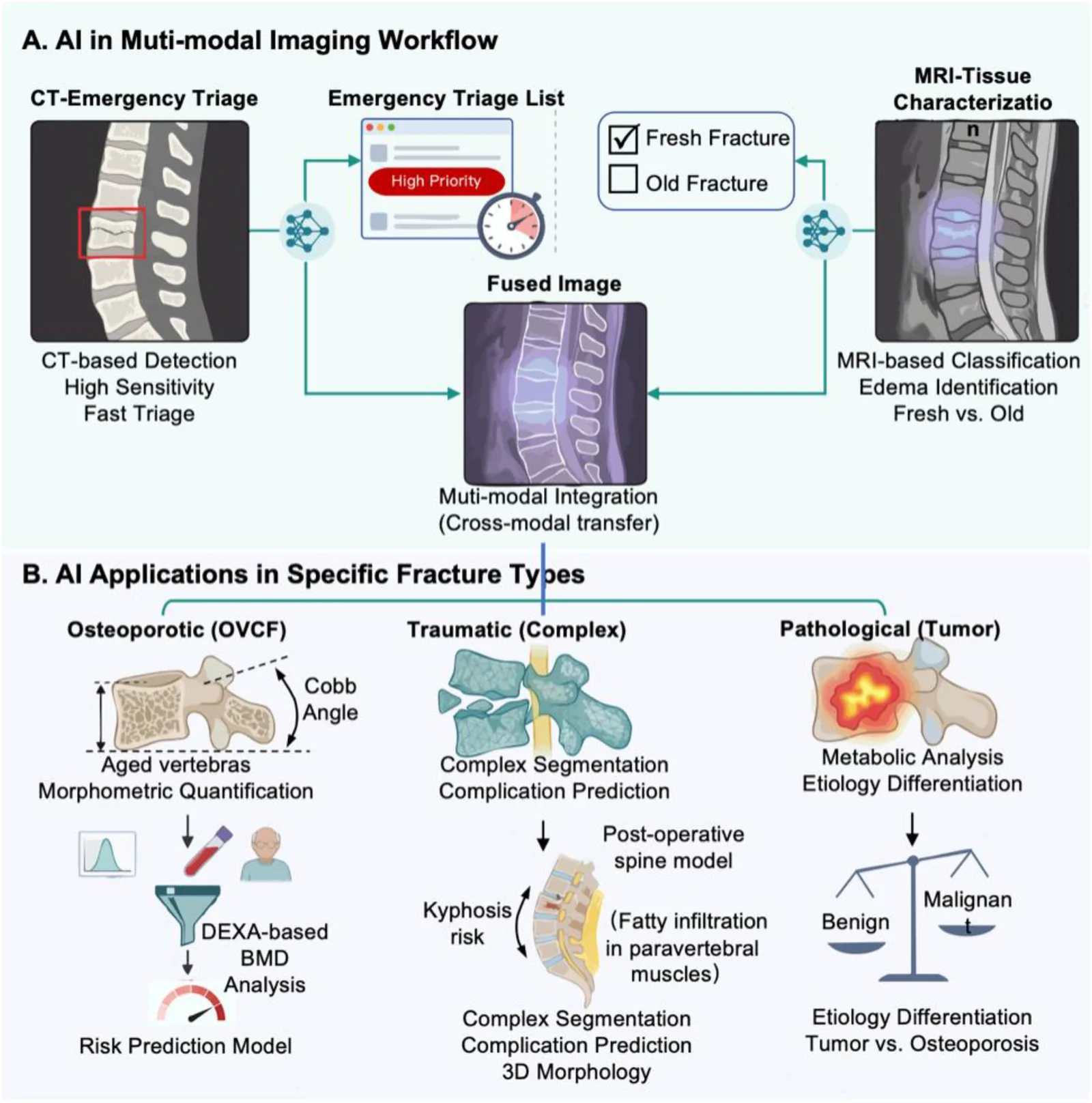
Panorama of multimodal AI applications in spinal fracture triage, subtype classification, and prognosis using CT, MRI, and DEXA. **(A)** AI in Multi-modal Imaging Workflow: CT-based emergency triage includes high-sensitivity detection and fused imaging, generating an emergency triage list (fresh vs. old fracture). MRI-based tissue characterization identifies edema and distinguishes fresh from old fractures, while multi-modal integration achieves cross-modal transfer. **(B)** AI Applications in Specific Fracture Types: For osteoporotic (OVCF) fractures, it includes morphometric quantification (e.g., Cobb angle) and DEXA-based BMD analysis for risk prediction modeling. Traumatic (complex) fractures involve complex segmentation, complication prediction (e.g., kyphosis risk), 3D morphology, and post-operative spine models assessing fatty infiltration in paravertebral muscles. Pathological (tumor) applications utilize metabolic analysis for etiology differentiation (benign vs. malignant, tumor vs. osteoporosis).

AI technologies now address the full clinical pathway: automated detection and classification using CT, MRI, and X-ray; differential diagnosis of osteoporotic, traumatic, and pathological fractures; surgical planning through 3D reconstruction and navigation; and prognosis prediction including complication risk and functional outcomes.

This review adopts a narrative synthesis rather than a formal systematic review or meta-analysis. This choice is based on three considerations: (i) the rapid evolution of AI architectures and the marked heterogeneity in training strategies and evaluation pipelines preclude meaningful quantitative pooling; (ii) performance metrics vary widely across studies owing to different fracture grading scales, reference standards, and threshold definitions, making direct statistical comparison inappropriate; and (iii) the field remains exploratory, where the priority is to map clinical applications and identify translational gaps rather than to derive a single accuracy estimate. Nevertheless, to minimise selection bias, we explicitly describe our search strategy, inclusion/exclusion criteria, and the limitations of the study selection process.

We searched PubMed, Web of Science, and Google Scholar for English-language studies published from January 2010 to June 2026 using combinations of the terms “artificial intelligence”, “deep learning”, “machine learning”, “spinal fracture”, “vertebral fracture”, “osteoporotic vertebral compression fracture”, “spinal imaging”, “CT”, “MRI”, “radiography”, “detection”, “classification”, “diagnosis”, “treatment planning”, and “prognosis”. Additional publications were identified through manual screening of reference lists from eligible articles and relevant reviews. We considered original studies, reviews, and meta-analyses addressing AI applications in spinal fracture imaging, diagnosis, treatment planning, or prognostic assessment. Case reports, conference abstracts, and non-English publications were excluded. Study selection was guided by relevance to the clinical questions of this review, with priority given to work reporting quantitative performance metrics, external validation, clinically meaningful comparators, or implementation-related findings. Consistent with a narrative review design, we did not perform a formal risk-of-bias assessment or meta-analysis.

## 2 Core technical foundations of AI in spinal fracture diagnosis

### 2.1 Machine learning and deep learning model architectures

Convolutional neural networks are central to current deep-learning approaches for spinal fracture imaging. By automatically extracting hierarchical features, they perform well in both fracture detection and segmentation tasks (24–26). U-Net, for instance, has become a standard architecture for medical image segmentation; one study integrating it with radiomics features achieved 95.7% accuracy in detecting odontoid process fractures (27). Residual Network (ResNet), which uses residual connections to counter gradient vanishing in deep networks, is well suited to classification; a recent study applying ResNet-18 with transfer learning showed strong performance in vertebral compression fracture classification (28). Given the variability in fracture location and morphology, multi-scale feature fusion networks have also been developed to improve segmentation of CT images, enabling better recognition of diverse fracture patterns (29). Taken together, these Convolutional neural networks (CNNs)-based architectures map three core technical pathways (encoder-decoder structures, residual learning, and multi-scale feature extraction) directly onto three clinical priorities: segmentation precision, model depth, and feature robustness.

Support vector machines (SVM) and other traditional machine learning methods still offer value, particularly when paired with handcrafted imaging features (24, 30). In one study, researchers used a Vision Transformer to extract features from x-rays and then applied SVM to identify spinal fractures involving posterior wall injury. The model achieved an area under the curve (AUC) of 0.909 at the training center and 0.837 at an external validation site, surpassing the performance of spine surgeons with three years of experience (31). This suggests that, for certain complex classification tasks, combining traditional classifiers with advanced feature extractors can yield both accuracy and interpretability. A similar pattern emerged in work on identifying the primary origin of spinal metastases, where models based on expert-defined imaging features outperformed pure deep learning approaches (32). Such findings point to a hybrid strategy that retains distinct advantages: it generalizes well even with limited training data and, just as critically, links each decision back to specific radiographic signs. That traceability creates a closed loop for clinical reasoning, an asset in medical contexts where data are scarce or the cost of misdiagnosis is high.

Object detection models like YOLO and Faster R-CNN can pinpoint fractured vertebrae directly in full CT or MRI images, offering one-step diagnosis (33). YOLO, in particular, has drawn attention for its speed: in one study, researchers paired YOLOv5m for vertebral detection with InceptionV3 to classify osteolytic metastases, creating a workable pipeline for rapid screening (34). Another group applied YOLOv8 to MRI scans for automated detection and classification of various vertebral lesions, reporting high mean average precision (18). These frameworks skip the need to analyze slice by slice; instead, they output fracture location and type from the whole image. That leap in automation makes them well suited to emergency settings or population-wide screening (35). What sets these object detectors apart is how they merge localization and classification (historically two separate steps) into a single, end-to-end process. The result is not only greater efficiency but also models that make fuller use of information contained in the lesion itself.

It is important to interpret such “superiority” claims with caution. Comparisons between AI and clinicians are often confounded by differences in reading conditions, test-set enrichment for obvious fractures, and non-independent reference standards. Moreover, many high-performing models are developed and tested on curated data from the same institution, leading to overfitting—a well-recognised issue in AI that is frequently downplayed in spinal fracture papers. For instance, the study cited above reported a substantial drop in AUC upon external validation, illustrating the fragility of performance estimates in real-world settings. Such degradation is not exceptional and underscores…before any claim of clinical superiority can be entertained. Moreover, direct comparisons between architectural families are effectively precluded by divergent datasets and evaluation protocols, leaving the question of optimal model selection empirically unresolved (Table 1).

**Table 1:** Representative AI architectures and their applications in spinal fracture imaging.

Model architecture	Core task	Technical features	Training strategy	Input data	Dataset size	Representative study	External validation	Reference
U-Net	Fracture segmentation and detection	Symmetric encoder-decoder structure, skip connections to preserve spatial information	End-to-end supervised learning, Dice loss function	Axial CT images of the dens axis	150 cases (75 fractures, 75 controls)	Detection of dens axis fractures	None reported	(27)
ResNet-18	Vertebral compression fracture classification	Residual connections, supports 152-layer deep network, alleviates vanishing gradient	Transfer learning (ImageNet pre-training), fine-tuning	Lateral radiographs	Multicenter: 907 training + 455 validation	Classification of vertebral compression fractures	Yes (multicenter validation cohort)	(28)
YOLOv8	Multi-type vertebral lesion detection and classification	Single-stage detector, CSPDarkNet backbone, anchor-free mechanism	Data augmentation (rotation, flipping, scaling), transfer learning	MRI T1/T2/STIR sequences	247 cases, augmented to 4,179 images	Classification of vertebral lesions (fractures, metastases, hemangiomas, etc.)	None reported	(18)
Vision Transformer + SVM	Prediction of spinal fractures with posterior wall injury	ViT self-attention mechanism extracts global features, SVM classifier	ViT pre-training + feature extraction, linear SVM	Lateral radiographs	Training center: 845 cases; validation center: 355 cases	Prediction of spinal fractures with posterior wall injury	Yes (independent external validation center)	(31)
Multi-scale feature fusion network	Automatic fracture segmentation on CT images	Parallel multi-scale convolution kernels, attention mechanism for feature fusion	End-to-end supervised learning, combined loss function	Spinal CT	Public dataset (VerSe2019) + internal data	Spinal fracture segmentation	Partial: public benchmark plus internal data; no fully independent clinical external cohort reported	(29)

### 2.2 Medical image preprocessing and data augmentation techniques

Image preprocessing helps bring out fracture contrast and highlight pathological features, both critical for boosting AI model performance (27, 36). In CT imaging, adjusting window level and width is a routine step for better visualization of bone and soft tissue. A study on odontoid process fractures, for example, found that a pipeline combining region-of-interest–based window optimization, U-Net segmentation, and radiomics features outperformed pure deep learning methods, pointing to the value of tailored preprocessing when fractures are subtle (27). For plain x-rays, preprocessing usually means contrast enhancement and noise reduction to clean up the input.

Beyond preprocessing, data augmentation has become a go-to strategy when labeled data are scarce (1, 37). Simple transformations (rotation, flipping, scaling) let researchers stretch existing datasets, giving models more room to generalize. The field now treats it as standard: in developing their MRI-based YOLOv8 model, one group used augmentation to generate 4,179 images for training and validation (18). Still, augmentation can only go so far. Building large, high-quality, multi-center annotated datasets remains the real bottleneck (1).

Spinal curvature adds another layer of complexity. Because the spine bends in both sagittal and coronal planes, vertebral alignment and shape vary widely, making detection and segmentation trickier. One solution is image reformatting: reconstructing a volume of interest along the spinal canal centerline to effectively “straighten” the spine (12, 38). This standardizes vertebral orientation, letting models focus on generic anatomical features rather than curve-induced variation. A study on automated vertebral fracture assessment in chest CT stopped short of an explicit straightening step, yet its network, built around vertebral likelihood maps and multi-parameter segmentation, handled native curved images well enough to hit a Dice score of 0.984, laying the groundwork for fracture evaluation (38). For multi-level assessment that is both automated and standardized, this kind of approach carries real promise.

## 3 AI applications in the diagnostic pathway for spinal fractures

Based on the core AI technologies outlined above, this chapter examines their clinical applications in spinal fracture imaging using a clinically oriented framework.

### 3.1 Automated detection and classification of common fracture types

#### 3.1.1 Osteoporotic vertebral compression fractures (OVCF)

In older adults, OVCF represent the most common fragility fractures, with bone mineral density decline as a well-established risk factor (39). For OVCF detection, AI models applied to CT images have demonstrated strong diagnostic performance (1, 16, 17). AI software has reached 89.6% accuracy in detecting moderate-to-severe vertebral compression fractures on CT, with specificity of 92.7% and a negative predictive value of 94.8%, helping non-specialist radiologists catch fractures they might otherwise overlook (40).

On plain radiographs, where OVCF signs can be subtle (particularly for low-grade compression fractures or vertebrae with severe degenerative changes), AI offers valuable decision support (5). A study of thoracolumbar x-rays reported specificity above 96% at the vertebral level for detecting OVCF; sensitivity for mild fractures still needs improvement, but the proof of concept is established (5). Reported CT superiority over X-ray for mild fractures may partly reflect differences in test-set difficulty rather than genuine model capability, as fracture-grade-specific performance is rarely reported across modalities.

In the MRI context, AI models built on architectures such as YOLOv8 can distinguish between acute and chronic OVCF using bone marrow edema patterns as the key discriminator (18, 41). One group trained a deep learning model to differentiate acute from old fractures on thoracolumbar CT images, using MRI-confirmed bone marrow edema as ground truth for acute fractures. This cross-modal transfer strategy, using MRI findings as training labels to guide CT image analysis, sidesteps the practical difficulty of perfectly registering multimodality data and points toward multimodal feature fusion within a single imaging modality (42).

Beyond detection, AI can precisely quantify vertebral height loss and wedge angle, enabling objective OVCF grading (43). This shift from qualitative to quantitative assessment makes diagnosis more objective and consistent, providing firmer imaging grounds for early intervention.

#### 3.1.2 Traumatic spinal fractures

For acute traumatic spinal fractures, CT remains the preferred imaging method due to its high spatial resolution and rapid acquisition (1, 44). CT-based AI models have shown strong performance in fracture detection, enabling rapid whole-spine screening and identification of subtle injuries (16, 45). One study framed fracture detection as a segmentation task using a U-Net architecture and reported 87.2% sensitivity for cervical spine fractures (12). Systems that automatically flag suspicious fractures for prioritization can reduce reading time and missed fractures (46, 47). One study found that AI-based prioritization shortened time to diagnosis for confirmed cervical fractures by 46% on average, approximately 16 min (46).

Performance, however, varies considerably. A meta-analysis estimated pooled sensitivity for spinal fracture detection at roughly 0.82, lower than that for skull, rib, or pelvic fractures (16). Another study noted that CNN sensitivity for cervical fractures ranged from 54.9% to 87.2% and dropped when differentiating old fractures (45). This wide variability likely arises from heterogeneous exclusion criteria and case composition rather than algorithmic differences alone; the consistently poorer performance on old fractures further suggests a lack of temporal understanding. This variability points to a deeper issue: current models struggle with morphological changes that occur as bones heal. Incorporating temporal dynamics into future architectures may improve diagnostic consistency over time.

For complex traumatic patterns such as burst fractures or fracture-dislocations, AI models are beginning to help identify key imaging features. Automated segmentation and three-dimensional morphological quantification now enable assessment of canal compromise and fragment displacement (48). In MRI, AI can clearly delineate bone marrow edema and soft tissue injury, providing critical information for trauma treatment decisions (41, 49).

Despite these limitations, AI has demonstrated value in clinical workflows, particularly in emergency settings. The evolving focus, from full automation to decision support, reflects a broader recognition of how AI best integrates into medical imaging. Human–machine collaboration, rather than replacement, enables smarter use of diagnostic resources, a benefit most evident in time-sensitive environments like emergency departments.

#### 3.1.3 Pathological vertebral fractures

MRI offers unique advantages in differentiating pathological from benign osteoporotic fractures: it provides imaging features essential for distinction, including bone marrow edema patterns, soft tissue characteristics, and morphological features of bone destruction (49). Using multiparametric data from T1, T2, and fat-suppressed sequences, AI models can separate osteoporotic compression fractures from spinal metastases (41). Deep learning and machine learning approaches have been applied to this differential diagnostic task with promising results (19). Such near-perfect results in this domain are not isolated and likely reflect overfitting to narrow datasets rather than genuine generalizability, pointing to a systemic overoptimism that warrants greater methodological scrutiny.

AI is proving useful for distinguishing among causes of pathological spinal fractures by applying deep learning to automatically assess patterns of bone destruction, lesion extent, and metabolic signals. A recent review noted that personalized numerical simulation and AI are emerging as tools for estimating fracture risk in bone metastases and myeloma (50). This points toward a necessary convergence: morphological description must be paired with biomechanical evaluation to quantify how tumor infiltration weakens vertebral structure.

In the MRI context, models built on architectures such as YOLOv8 can distinguish between acute compression fractures, metastases, hemangiomas, and spondylitis, with classification accuracy exceeding 0.84 (18). A similar pattern emerged in work identifying primary origins of spinal metastases, where models based on expert-defined imaging features outperformed pure deep learning approaches (32). Such findings suggest a hybrid strategy that generalizes well even with limited training data and, critically, links each decision back to specific radiographic signs.

Integrating AI with positron emission tomography–computed tomography (PET-CT) or whole-body MRI could enable end-to-end analysis of neoplastic spinal disease. By flagging abnormal metabolic activity or signal changes, AI might help locate occult primary tumors at a critical point in the diagnostic pathway. Direct evidence for distinguishing spinal infection from fracture remains limited, but AI's ability to parse bone destruction patterns and soft tissue features has laid groundwork. Collectively, these developments suggest AI is stitching together a diagnostic pipeline that runs from focal detection to whole-body screening, pushing etiological differentiation beyond single-image comparison toward a multi-level framework that detects, characterizes, and traces lesions to their source.

### 3.2 Granular quantitative analysis and risk stratification

#### 3.2.1 Automated morphological quantification

AI demonstrates clear strengths in automated measurement and quantitative analysis for spinal imaging. Deep learning models can now segment vertebrae, discs, and the spinal canal with high precision, generating objective metrics rapidly (51). For scoliosis or post-fracture deformity, they automatically calculate Cobb angles with accuracy validated across multiple studies (52). In spinal stenosis assessment, AI systems segment canal boundaries and compute cross-sectional area with less subjectivity and shorter time than manual measurement (20). The net effect: spinal imaging is moving away from semi-quantitative visual estimates toward fully quantitative parameter measurement.

For fracture assessment, AI can extract morphological data (vertebral body height and related parameters) and apply the Genant scale to automatically detect and grade compression fractures (38). A study of thoracolumbar x-rays reported specificity above 96% at the vertebral level for automated Genant grading (5). Beyond measurement, AI can package these metrics into structured reports that include fracture location, type, grade, and key numerical values. This standardization makes reporting more consistent and comprehensive, allowing clinicians to see essential information at a glance (1).

#### 3.2.2 AI-Based fracture risk prediction

The structured quantitative metrics produced by automated segmentation (height, wedge angle, canal area) directly feed into risk prediction models. When estimating fracture risk, AI's ability to integrate these imaging-derived parameters with clinical and laboratory data provides a more complete picture than any single modality alone. For OVCF, bone mineral density is central (53, 54), but age ≥70, female sex, diabetes, prior non-spine fractures, and vitamin D deficiency all contribute independently (39, 55, 56). Machine learning can incorporate bone metabolism markers, inflammatory indicators, and sarcopenia to predict OVCF risk in postmenopausal osteoporotic women, with reported accuracy as high as 98.98%—a step toward truly individualized risk stratification (53). However, this near-perfect accuracy figure originates from a single-center retrospective study and should be interpreted with caution. The absence of external validation and reported confidence intervals raises concerns about potential overfitting and data leakage. Beyond this specific example, such performance inflation is not isolated; many studies in this field share similar methodological weaknesses—including selective reporting of favourable metrics, exclusion of challenging cases, and lack of pre-registered analysis plans—which collectively limit real-world applicability. Future work must validate such models in independent, prospectively collected cohorts before clinical adoption. One model built on age, height loss, and femoral neck T-scores performed comparably to the traditional Fracture Risk Assessment Tool (FRAX) tool, suggesting a new avenue for fracture risk screening in asymptomatic populations (43). The broader implication is that OVCF reflects systemic decline, not merely local bone failure. By surfacing nonlinear interactions among clinical, imaging, and laboratory variables, AI offers clinicians a new approach to parsing risk in multifactorial disease.

For spinal metastases, AI combined with quantitative CT biomechanical simulation can assess pathological fracture risk on a case-by-case basis, informing decisions about activity and rehabilitation (20, 50). This extends risk prediction from primary osteoporotic fractures to neoplastic bone disease, demonstrating the versatility of AI-based approaches.

## 4 AI in treatment planning and prognostic prediction for spinal fractures

Beyond diagnosis, AI is increasingly integrated into therapeutic decision-making and outcome prediction for spinal fractures. This chapter extends the discussion from imaging-based diagnosis to treatment-oriented applications (Figure 2).

**Figure 2:**
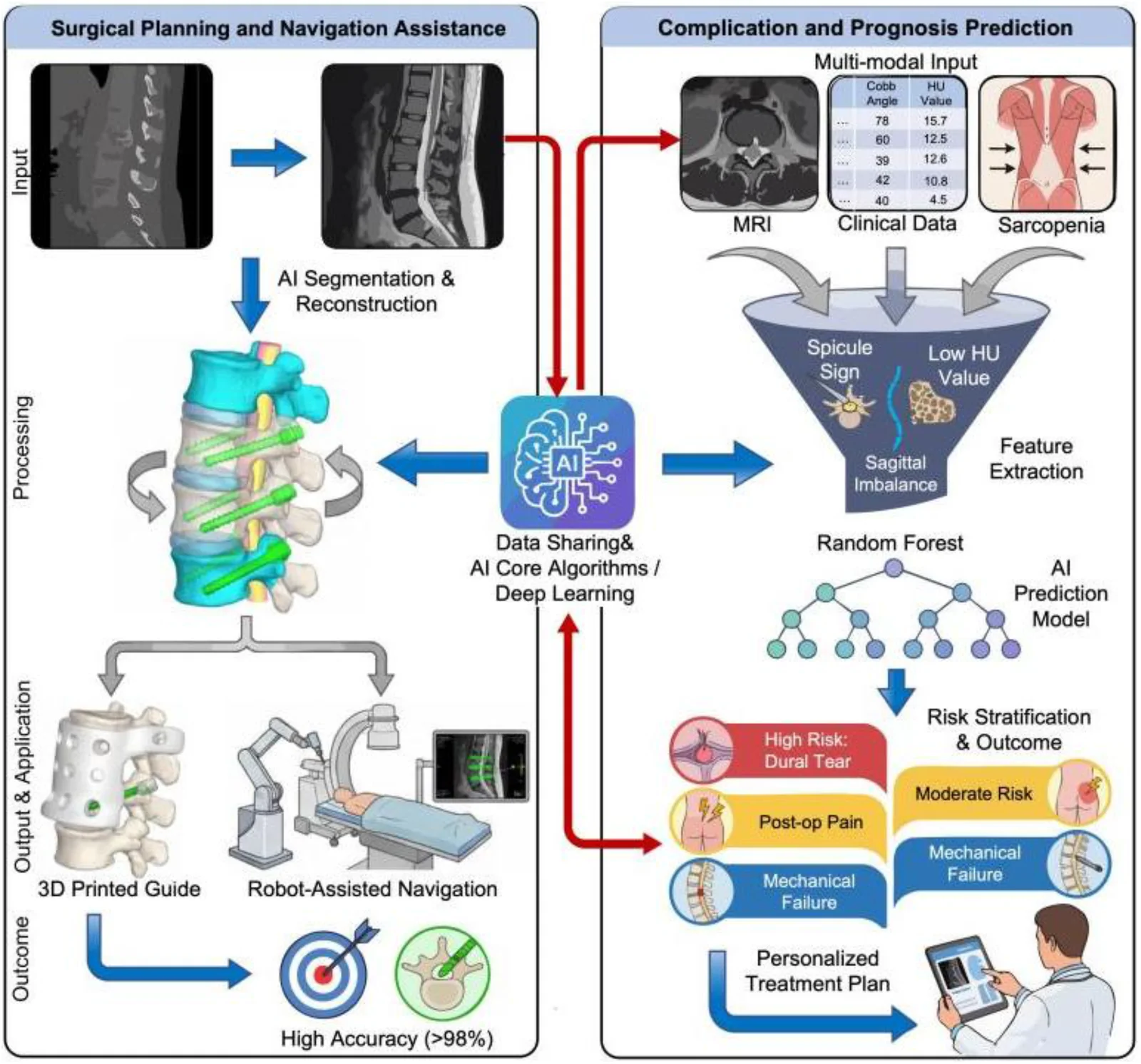
AI-powered workflow for surgical planning and prognostic prediction in spinal fractures. The surgical planning module segments CT/MRI data to generate patient-specific 3D models and screw trajectories, supported by 3D-printed guides or robotic navigation for precise placement. The prognostic module integrates multimodal inputs (MRI, clinical data, sarcopenia), extracts key imaging features (spicule sign, low HU value, sagittal imbalance), and applies machine learning (e.g., random forest) to predict complications (mechanical failure, postoperative pain, dural tear) and stratify risk, thereby enabling personalised treatment plans. Both modules share core deep-learning algorithms and data-sharing infrastructure, forming a closed-loop decision-support system with consistently high predictive accuracy.

### 4.1 Surgical planning and navigation assistance

AI is now being woven into technologies such as three-dimensional reconstruction and augmented reality, collectively pushing spinal fracture surgical planning toward greater precision (57). AI algorithms take preoperative CT or MRI scans, automatically segment vertebrae and pedicles, and build patient-specific 3D models, work previously performed manually that now feeds directly into screw trajectory planning (51, 58–61). The evidence supports this approach: one study of upper thoracic fracture surgery found that 3D-printed screw guides based on CT data improved accuracy to 96.6%, compared with 83.6% for freehand placement (62). AI can also generate synthetic MRI-like images from CT, potentially reducing the need for multiple scans and saving time and cost (20, 63).

In the operating room, AI-equipped robotic systems paired with image guidance are making screw placement safer and more accurate. A large retrospective review of over 5,400 thoracolumbar pedicle screws placed with O-arm navigation reported 98.63% accuracy, a major breach rate of only 0.55%, and no screw-related neurovascular injuries (64). In challenging areas like the cervical spine (where anatomy can vary considerably, as in rheumatoid arthritis patients), CT-based navigation or microscope-integrated augmented reality ensures accurate C1-C2 screw placement (65). These systems can add time and radiation exposure due to repeated intraoperative imaging (66). However, for complex cases, unstable pelvic ring fractures or fractures in ankylosing spondylitis, the gains in accuracy and reduction in complications justify the trade-off (67, 68).

### 4.2 Prediction of complications and prognosis

#### 4.2.1 Postoperative mechanical complications

The predictive factors and outcome definitions vary substantially across studies, and most models lack independent external validation, substantially limiting their generalisability. Nevertheless, AI is proving useful for predicting postoperative mechanical complications by extracting personalized risk estimates from preoperative images and clinical data (51, 69–71). For OVCF patients, complications after vertebral augmentation (cement displacement, vertebral re-collapse, new adjacent fractures) are more likely when fractures occur at the thoracolumbar junction, when preoperative vacuum clefts are present, when cement distributes in clumps, when anti-osteoporosis therapy is inconsistent, or when paraspinal muscle fatty infiltration is severe (72–74). Nomograms incorporating these factors can identify high-risk patients before surgery, helping tailor both surgical approach and postoperative care (75).

For implant-related mechanical problems, CT Hounsfield units provide a straightforward quantitative metric. A multicenter study found that low preoperative lumbar HU values and global sagittal imbalance independently identified patients at risk for complications after adult spinal deformity surgery (76). Researchers have also focused on adjacent segment fracture after vertebroplasty and progression of kyphotic deformity. Consistently identified factors include preoperative kyphosis angle, endplate injury, paraspinal muscle fatty infiltration, and cement distribution patterns (77–79). These studies share a common goal: integrating discrete imaging signs and surgical variables into a single quantitative framework, moving risk assessment from single-factor heuristics toward data-driven stratification.

Machine learning models are increasingly replacing traditional statistical approaches here. One SVM-based model predicts new OVCF risk and has been incorporated into a clinical calculator (80). This trend reflects a broader shift: by uncovering nonlinear relationships between deep imaging features and clinical outcomes, AI is helping transform postoperative management from reactive to proactive.

#### 4.2.2 Clinical outcomes and functional prognosis

Beyond mechanical complications, AI can predict patient-centered outcomes that directly impact quality of life. For OVCF patients, a random forest model predicted residual distal lumbosacral pain after percutaneous kyphoplasty (PKP) reasonably well, with preoperative Cobb angle and sarcopenia emerging as the two most important features (81). Another study using a Tabular Network (TabNet) model combining radiomics and clinical parameters achieved strong performance in predicting residual back pain after surgery (82).

In spinal metastases, AI combined with quantitative CT biomechanical simulation can assess pathological fracture risk on a case-by-case basis, informing decisions about activity levels and rehabilitation protocols (20). What ties these examples together is AI's ability to extract prognostically significant imaging features that are easily missed by the naked eye. This closes the loop: preoperative imaging features feed into postoperative outcome predictions, with surgical variables and patient characteristics analyzed within a single framework. In this way, AI moves beyond diagnosis to become a decision-support tool spanning the entire care pathway.

#### 4.2.3 Intraoperative complication warning

AI also helps identify high-risk injuries before patients reach the operating room. In lumbar burst fractures, for instance, researchers identified a “dural sac spike sign” on MRI that may help identify patients at increased risk of intraoperative dural tears (83). However, the reported sensitivity of 76% means that one in four such tears would be missed if this sign were used as the sole criterion; therefore, it should be viewed as an adjunctive warning sign rather than a reliable standalone predictor. Another study linked preoperative neurological deficits, widened interpedicular distance, severe canal compromise, and a laminar fracture gap >2 mm to traumatic dural tears in thoracolumbar fractures (84). Together, these AI-driven models and imaging biomarkers allow surgeons to enter the operating room with clearer risk awareness, tailor their approach accordingly, and potentially prevent secondary surgeries (Table 2).

**Table 2:** Summary of AI applications in prognostic prediction for spinal fractures.

Prediction target	Study design	Sample	Model(s)	Input features	Key predictors	Performance	Clinical implication	External validation	References
Residual distal lumbosacral pain after PKP	Retrospective cohort	386	Random forest, XGBoost, logistic regression	Cobb angle, vertebral height, age, BMD, paraspinal muscle area	Cobb angle >15°, sarcopenia, VAS >7	AUC 0.87, accuracy 82.3%	Preoperative risk stratification	No (internal split)	(81)
Mechanical complications after ASD surgery	Multicenter retrospective	289	Logistic regression, Cox regression	Lumbar HU, PI-LL mismatch, SVA, fusion length	HU <110, PI-LL >10°, age >65	AUC 0.84, sens 81%, spec 79%	Preoperative implant failure risk assessment	Yes (multicenter)	(76)
Intraoperative dural tear in lumbar burst fractures	Retrospective case-control	212 (42 with tear)	Imaging marker + logistic regression	Spiking sign, canal encroachment, lamina fracture, interpedicular distance	Spiking sign (OR 8.2), laminar gap >2 mm (OR 4.5)	Sens 76%, spec 91%*	Preoperative warning to improve safety	None	(83)
New OVCF after vertebroplasty	Retrospective cohort	534	SVM, logistic regression, nomogram	Age, sex, diabetes, BMD, cleft sign, cement distribution	BMD T-score <−2.5, cleft sign, lumpy cement	AUC 0.89, accuracy 85.3%	Individualized follow-up planning	Limited (temporal split)	(80)

## 5 Current challenges and limitations in clinical translation

Before examining specific technical barriers, we summarise the recurring methodological limitations across the studies included in this review. Although we did not apply a formal quality-scoring tool such as QUADAS-2—consistent with the narrative design—several systematic biases are evident. Most models were developed on single-centre retrospective cohorts; external validation, when performed, consistently yielded lower performance than internal testing. Non-independent reference standards, selective reporting of favourable metrics, and absence of pre-registered analysis plans were common, increasing overfitting risk. We have highlighted these issues in the relevant sections (see Tables 1, 2), and caution readers against interpreting reported accuracy as clinically established. Real-world deployment faces additional barriers—regulatory approval, interoperability, cost-effectiveness, clinician acceptance, and prospective validation—that remain understudied in spinal fracture AI.

### 5.1 Data quality and model generalizability

How well an AI model performs depends on the quantity, quality, and diversity of the data used for development and evaluation. At present, most spinal fracture studies still rely on retrospective, single-center datasets with heterogeneous annotation rules and limited external testing, which weakens confidence in generalizability (1). A related literature gap is that recent foundation-model paradigms are only beginning to enter spine imaging. Promptable segmentation systems such as SAM and MedSAM have shown feasibility for lumbar spine MRI, but their performance still lags behind task-specific supervised models, indicating that zero-shot transfer alone is not yet sufficient for dependable spinal image analysis (85). Likewise, broader vision-language and foundation-model frameworks may eventually support multimodal reasoning and cross-task adaptation in radiology, but they remain largely underexplored in spinal fracture workflows (86, 87).

Spinal imaging brings its own complications. Vertebral shapes and fracture types vary widely; different scanners, protocols, and reconstruction algorithms also leave distinct fingerprints on images. These factors collectively hinder model generalization (88). Annotations depend on radiologists' judgment, and variability (between readers, or even the same reader on different days) can steer a model toward biased features (89). Evidence shows that once data perturbations cross a certain threshold, predictive performance can tank or vanish entirely (90). The deeper problem is that many current models latch onto superficial statistical patterns in the training set rather than learning what a fracture actually looks like pathologically. So when the image style shifts even slightly, they stumble. Building large-scale, multi-center spinal fracture databases with clean, consistent annotations, and leveraging data augmentation and synthesis techniques, is a logical next step if we want models that generalise reliably (20, 91–93). However, data quantity alone is insufficient; rigorous methodological practices, including predefined exclusion criteria, blinding of reference standard readers, and adherence to reporting guidelines such as TRIPOD-AI, are equally critical to reduce bias and improve reproducibility. Fewer than half of surveyed studies fully report these safeguards, and prospective validation remains exceptionally rare (94).

### 5.2 Algorithm “black Box” and clinical interpretability

A persistent criticism of deep learning models is that their decisions are hard to trace (the “black box” problem), and this opacity keeps many clinicians from trusting or adopting them (1, 95–97). In spinal fracture diagnosis, it is not enough to know whether a model flags a fracture; the clinician needs to understand why. Without that insight, there is no easy way to double-check the model's reasoning, and integration into real-world workflows stalls (98, 99). Interpretability, in other words, is not a nice-to-have; it is a prerequisite for AI to earn a place in clinical decision-making.

Explainable AI (XAI) has become a lively research area as a result (96, 100). One common approach, gradient-weighted class activation mapping (Grad-CAM), produces heatmaps that show which parts of an image drove the model's decision, something a clinician can look at and evaluate (101). SHAP (SHapley Additive exPlanations) goes a step further, attaching numbers to each feature's contribution and giving the output a sense of accountability (101, 102). More recently, researchers have started building interpretability directly into models rather than adding it after the fact. One group, for instance, generated “pseudo-healthy” vertebral images and graded OVCFs by measuring relative height loss; because the decision hinges on quantifiable morphological change, it comes with a built-in pathophysiological explanation (103). What this evolution points to is a shift: from methods that simply point to where a model looked, toward architectures that align their internal logic with how clinicians actually think. Advancing explainable AI is therefore not just a technical challenge but a necessary step toward building trust and ensuring that AI-assisted diagnosis stays safe and accountable. Clinician acceptance is not automatic—systematic reviews identify lack of trust, unfamiliarity, and perceived threats to professional autonomy as primary barriers (104).

### 5.3 Clinical integration, ethical, and regulatory hurdles

Getting AI tools to actually work inside a hospital, plugged into the real systems radiologists and surgeons use every day, is harder than it looks. On the technical side, AI has to talk to Picture Archiving and Communication System (PACS), Radiology Information System (RIS), and Electronic Health Record (EHR), which means wrestling with data formats, building interfaces, and figuring out where the model should live (20, 105). On the administrative side, workflows have to shift, screens have to make sense to users, and someone has to decide who reviews the AI's output and who answers if something goes wrong. Interoperability with PACS and RIS remains a major technical hurdle, and cost-effectiveness evidence for spinal fracture AI is virtually absent, despite demonstrations of economic value in other radiology screening contexts (106).

Deeper down, the obstacles are ethical and regulatory. If an AI assists with a diagnosis and the patient is harmed, who carries the liability? The developer? The hospital? The doctor who signed off? The law has not caught up. Data privacy is another non-negotiable: how to collect, anonymize, share, and use patient data without crossing legal lines still needs clear rules (107–109). Bias is just as worrying. Train a model mostly on one population, and its performance will slip in another, potentially deepening inequities. Regulatory approval also needs to be discussed in more concrete product terms. Beyond general references to the FDA, clinically deployed spine-related tools already exist, including FDA-cleared cervical-spine fracture triage software from Aidoc and vertebral compression fracture detection software from Zebra Medical Vision, which illustrate that commercialization is feasible but typically limited to narrow, well-defined use cases rather than comprehensive fracture management (110, 111). The FDA review process for radiologic AI algorithms has unique challenges (112), yet post-market surveillance data for cleared products remain scarce (113).

Moving AI from promising papers to bedside reality will take more than better algorithms. Implementation barriers span operational, technical, clinical, and regulatory domains (114).

### 5.4 Special considerations in older adults

Older adults account for the highest burden of vertebral fractures, yet they remain a classic underdiagnosed population in routine practice. Vertebral fractures are frequently missed on radiographs, especially when patients are scanned for other indications or when symptoms are attributed to aging, degeneration, or frailty rather than fracture (7). This geriatric context matters because delayed recognition can postpone osteoporosis treatment, fall prevention, and secondary fracture prevention.

In older spines, multilevel degeneration, osteophytes, scoliosis, prior compression deformities, and diffuse bone loss can all interfere with image interpretation and reduce the specificity of AI systems trained on cleaner datasets (111, 115). Degenerative change may obscure vertebral endplate disruption or height loss, while osteopenia can flatten the visual contrast between normal and fractured vertebrae. For this reason, AI models intended for older adults should be validated specifically in aged cohorts and in imaging conditions that reflect common real-world comorbidity patterns.

Polypharmacy is another important geriatric issue. Medications such as glucocorticoids and proton pump inhibitors are associated with secondary osteoporosis or altered bone health, which may change fracture risk and complicate the clinical background against which images are interpreted (116, 117). In practice, medication exposure may not directly alter pixel-level image appearance, but it can change bone quality, fracture susceptibility, and the pre-test probability of vertebral collapse. Therefore, medication history should be considered when AI systems are deployed in older populations.

Frailty is increasingly recognized as a clinically meaningful input variable for AI-based risk stratification. Recent work has shown that machine learning methods can identify frailty in hospitalized older patients using readily available electronic health record variables, and frailty modeling studies suggest that functional performance, diagnosis patterns, and impaired cognition are among the most informative predictors (118, 119). In spinal care, frailty tools have also been reviewed as potentially relevant for risk stratification and perioperative planning (120). These findings support incorporating frailty indices or frailty-related features into AI pipelines when the goal is to estimate fracture risk, treatment tolerance, or recovery potential in elderly patients.

Cognitive impairment adds a separate layer of complexity because AI-assisted diagnosis and treatment still depend on valid informed consent. In older adults with cognitive impairment, decision-making capacity may be reduced and should be assessed carefully, especially when surrogate decision makers or substituted judgment are required (121, 122). In parallel, patients increasingly expect disclosure when AI contributes to diagnosis, and their preferences for information vary with age and other characteristics (123). Accordingly, AI deployment in geriatric spine care should include capacity assessment, disclosure policies, and workflow safeguards that respect autonomy while preserving timely care.

Validation in older adults should not be treated as an afterthought. Studies directly evaluating AI for osteoporotic vertebral fracture detection in post-menopausal or older populations have shown that performance can be good, but sensitivity may still vary by fracture severity, imaging modality, and the ground truth used for training and validation (124). More broadly, a recent meta-analysis confirmed that machine learning can achieve high diagnostic accuracy for vertebral fractures, but also emphasized the need for further validation across populations and imaging contexts (125). For older adults, this means that model testing should report age-specific performance, spectrum of degeneration, medication burden, frailty status, and cognitive status whenever possible. Most current AI studies either exclude older adults with significant comorbidities or fail to report subgroup analyses by age, making it impossible to assess generalizability to the population that stands to benefit most (126).

## 6 Future research directions and prospects

### 6.1 Integration of multimodal and multi-center data

If AI is going to make a real difference in spinal fracture care, it needs data: lots of it, and the right kind. Large-scale, multi-center, multi-modal imaging databases are the necessary foundation. Right now, most models train on a single modality (CT, MRI, or x-ray), but each tells only part of the story. CT shows bone well; MRI is better for soft tissue (127, 128). Marry the two, pull in cases from multiple centers, and you give a model half a chance at being both robust and generalizable (129). Public benchmarks also deserve a more explicit role in this discussion. In particular, the VerSe challenge has become a key reference standard for vertebral labeling and segmentation across multi-center CT datasets, providing a shared benchmark for comparing algorithm performance and stress-testing model robustness under anatomical variation and domain shift (130). Although VerSe is not a fracture-specific benchmark, its public availability and standardized evaluation framework make it highly relevant for technical comparison and reproducibility in spine AI research.

Further out, there is talk of bringing in multi-omics: genomics, proteomics, the whole molecular picture. In other fields (pancreatic cancer, glioblastoma), models that fuse imaging with molecular data have already beaten traditional approaches on prognosis (131, 132). For spinal fractures, the hope is that the same strategy could sharpen fracture classification, stability assessment, and maybe even prediction of bone healing or neurological recovery. That is the personalized-medicine pitch. But it is also expensive, analytically brutal, and no one has quite figured out how to fuse omics data with imaging features cleanly. Those are the questions that will keep the field busy for the next decade.

### 6.2 Advanced models and learning paradigms

If spinal fracture diagnosis is to move forward, it will need to look beyond standard convolutional architectures. Newer designs (Transformers, vision-language models) handle long-range dependencies and context better. Beyond CNNs, foundation models (e.g., SAM, MedSAM) and vision-language architectures are emerging in spine imaging, but their zero-shot performance on spinal CT/MRI remains inferior to task-specific models, and multimodal LLMs that integrate imaging with clinical text face hurdles of scarce paired datasets and domain shift (85–87). Systematic evaluation of these paradigms on spine-specific benchmarks is urgently needed before clinical translation. That could sharpen detection of complex fracture patterns, improve assessment of canal involvement, and refine differential diagnosis (129). They have already made a difference in coronary imaging; there is no reason spinal imaging cannot benefit in the same way: finer detection, more nuanced classification, richer quantification (133).

Two hard constraints never go away: labeled data are scarce, and privacy matters. That is where alternative learning paradigms come in. Few-shot learning stretches small datasets further, useful for rare fractures (134). Self-supervised learning taps unlabeled images for pre-training, cutting the need for manual annotation. Federated learning lets institutions collaborate without moving data off-site, pooling knowledge while keeping records local (129). Spine surgery has already seen work that integrates EHR data with multimodal machine learning to predict perioperative safety (135). Marry these paradigms with the new architectures, and you get diagnostic tools that are not only more powerful but also built to work within real-world limits.

### 6.3 Toward standardized clinical validation and implementation

AI in spinal fracture imaging only proves its worth when it has been put through rigorous clinical testing and shown to work safely in the real world. Prospective, multicenter randomized controlled trials (RCTs) are the gold standard here. Most studies so far stop at retrospective analysis and internal checks; we still lack the high-grade evidence that AI actually improves what matters to patients (129). That means designing RCTs that compare AI-assisted diagnosis with the usual approach, and looking beyond simple accuracy, at how AI changes decisions, shifts resource use, and affects long-term functional recovery (136).

Making sure these tools are safe, fair, and effective will take more than good algorithms. It needs teams that bring together radiologists, orthopedic surgeons, neurosurgeons, computer scientists, and ethicists (129). Those teams should hammer out guidelines, set standards, and build training programs. Guidelines need to spell out when to use AI, how to read its output, and who does what. Standards should cover data quality, model validation, regulatory oversight, and ongoing monitoring. Training clinicians systematically, so they grasp what the tool does, where it falls short, and how it might be biased, is the only way to get real uptake. Ophthalmology has already started working through these questions with ChatGPT-based multimodal systems; their experience offers a roadmap (137). Without that kind of rigor, AI risks staying a promising idea rather than becoming a reliable part of care. For future evidence syntheses, we encourage a shift from narrative summaries to systematic review methodologies, including pre-registered protocols, comprehensive search strategies, and formal bias assessment using validated tools. Such an approach would provide more reliable conclusions and better inform clinical practice.

## 7 Conclusions

AI in spinal fracture imaging has moved past the proof-of-concept stage and is now feeling its way toward real clinical use. On CT and MRI, models can already detect and classify osteoporotic, traumatic, and metastatic fractures with enough reliability to cut reading time and smooth out disagreements between observers. That matters. And the scope is widening: what started as a diagnostic assist is reaching into treatment planning and prognosis, potentially a tool that stays with the patient from the first scan through recovery.

Nevertheless, the accumulated evidence reveals a clear translational gap. While technical performance in controlled, single-centre settings is often impressive, it consistently overestimates real-world utility. Performance deteriorates for mild fractures, degenerated spines, and postoperative anatomy; external validation remains the exception rather than the rule; and the few commercially cleared products lack independent peer-reviewed scrutiny. These patterns reflect a systemic bias toward publishing positive results from curated datasets while neglecting the heterogeneous data that constitute routine clinical practice.

This bias is compounded by the frequent absence of critical methodological appraisal in review articles, which risks perpetuating overoptimistic interpretations. We therefore call for future reviews to adopt structured quality assessment and for primary studies to report pre-specified analysis plans and handling of missing data.

Looking forward, we see three priority actions that would substantively alter the trajectory. First, the spine imaging community should adopt public benchmarks (such as VerSe) and standardised reporting checklists (such as CLAIM) as mandatory requirements for model publication, enabling fair cross-study comparison and exposing overfitting. Second, researchers must move beyond retrospective case-control designs toward prospective, multi-centre registries that capture the full spectrum of fracture types, patient demographics, and imaging hardware, with predefined validation splits and analysis plans. Third, and most importantly, the field needs randomised controlled trials that measure patient-centred outcomes—pain, mobility, quality of life, and return to function—rather than surrogate metrics like AUC or sensitivity. These trials should also evaluate human-AI interaction, because the ultimate benefit of AI depends not on its standalone accuracy but on whether it improves clinical decisions when used as intended.

Achieving these goals will require sustained collaboration among radiologists, surgeons, data scientists, regulators, and patients. It will also require a cultural shift: away from treating AI as a magic bullet, and toward treating it as a tool that must be rigorously tested, transparently reported, and continuously monitored in the wild. The promise is real, but the path to reliable patient benefit is paved with evidence, not enthusiasm.

## References

[1] Jeong S Lee BJ. Advancing spine fracture detection: the role of artificial intelligence in clinical practice. Korean J Neurotrauma. (2025) 21:172–82. 10.13004/kjnt.2025.21.e22 40778250 PMC12325887

[2] Alhadhoud M Alsiri N. The epidemiology of spinal fractures in a level 2 trauma center in Kuwait. SAGE Open Med. (2021) 9:20503121211051932. 10.1177/20503121211051932 34671474 PMC8521410

[3] Roth S Oberthür S Sehmisch S Decker S. Osteoporotic vertebral fractures of the thoracic and lumbar spine. Unfallchirurgie (Heidelb). (2024) 127:263–72. 10.1007/s00113-023-01407-9 38276974

[4] Girdler SJ Lindsey MH Sebastian AS Nassr A. Osteoporosis evaluation and management in spine surgery. J Am Acad Orthop Surg. (2024) 32:e909–18. 10.5435/jaaos-d-24-00311 39083525

[5] Oppenheimer J Lüken S Geveshausen S Hamm B Niehues SM. An overview of the performance of AI in fracture detection in lumbar and thoracic spine radiographs on a per vertebra basis. Skeletal Radiol. (2024) 53:1563–71. 10.1007/s00256-024-04626-2 38413400 PMC11194188

[6] López Zúñiga D Láinez-Ramos-Bossini AJ Ruiz Santiago F. Radiographic diagnosis of osteoporotic vertebral fractures. An updated review. Med Clin (Barc). (2022) 158:125–32. 10.1016/j.medcli.2021.06.019 34392986

[7] Li Y Yan L Cai S Wang P Zhuang H Yu H. The prevalence and under-diagnosis of vertebral fractures on chest radiograph. BMC Musculoskelet Disord. (2018) 19:235. 10.1186/s12891-018-2171-y 30021567 PMC6052683

[8] Fathi M Eshraghi R Behzad S Tavasol A Bahrami A Tafazolimoghadam A. Potential strength and weakness of artificial intelligence integration in emergency radiology: a review of diagnostic utilizations and applications in patient care optimization. Emerg Radiol. (2024) 31:887–901. 10.1007/s10140-024-02278-2 39190230

[9] Kwee RM Toxopeus R Kwee TC. Imaging overuse in the emergency department: the view of radiologists and emergency physicians. Eur J Radiol. (2024) 176:111536. 10.1016/j.ejrad.2024.111536 38820950

[10] Chen J Gandomkar Z Reed WM. Investigating the impact of cognitive biases in radiologists’ image interpretation: a scoping review. Eur J Radiol. (2023) 166:111013. 10.1016/j.ejrad.2023.111013 37541180

[11] Zhan H Schartz K Zygmont ME Johnson JO Krupinski EA. The impact of fatigue on Complex CT case interpretation by radiology residents. Acad Radiol. (2021) 28:424–32. 10.1016/j.acra.2020.06.005 32622748

[12] Golla A-K Lorenz C Buerger C Lossau T Klinder T Mutze S. Cervical spine fracture detection in computed tomography using convolutional neural networks. Phys Med Biol. (2023) 68:115010. 10.1088/1361-6560/acd48b 37167980

[13] Zhou X Wang H Feng C Xu R He Y Li L. Emerging applications of deep learning in bone tumors: current advances and challenges. Front Oncol. (2022) 12:908873. 10.3389/fonc.2022.908873 35928860 PMC9345628

[14] Ounasser N Rhanoui M Mikram M El Asri B. Deep learning-based anomaly detection in orthopedic medical imaging: a systematic literature review. J Orthop. (2025) 69:329–45. 10.1016/j.jor.2025.07.015 40895636 PMC12398233

[15] Gu Y Wang Y Li M Wang R. Current applications of deep learning in vertebral fracture diagnosis. Osteoporos Int. (2025) 36:2071–82. 10.1007/s00198-025-07604-z 40764417

[16] Husarek J Fuchss AMC Ruder TD Mougiakakou S Exadaktylos A Wahedi K. Artificial intelligence for fracture detection on computed tomography: a comprehensive systematic review and meta-analysis of diagnostic test accuracy in non-commercial and commercial solutions. Emerg Radiol. (2026) 33:351–75. 10.1007/s10140-026-02437-7 41652293 PMC13079534

[17] Spångeus A Bjerner T Lindblom M Götz C Hummer A Salzlechner C. Breaking the silence: aI’s contribution to detecting vertebral fractures in opportunistic CT scans in the elderly-a validation study. Arch Osteoporos. (2025) 20:42. 10.1007/s11657-025-01524-5 PMC11937205 40131601

[18] Er H Tören M Asan B Kaba E Beyazal M. Deep learning-based differentiation of vertebral body lesions on magnetic resonance imaging. Diagnostics (Basel). (2025) 15:1862. 10.3390/diagnostics15151862 40804827 PMC12345889

[19] Jeong W Baek C-H Lee D-Y Song S-Y Na J-B Hidayat MS. The classification of metastatic spine cancer and spinal compression fractures by using CNN and SVM techniques. Bioengineering (Basel). (2024) 11:1264. 10.3390/bioengineering11121264 39768082 PMC11673390

[20] Lee S Jung JY Mahatthanatrakul A Kim JS. Artificial intelligence in spinal imaging and patient care: a review of recent advances. Neurospine. (2024) 21:474–86. 10.14245/ns.2448388.194 38955525 PMC11224760

[21] Franco PN Maino C Mariani I Gandola DG Sala D Bologna M. Diagnostic performance of an AI algorithm for the detection of appendicular bone fractures in pediatric patients. Eur J Radiol. (2024) 178:111637. 10.1016/j.ejrad.2024.111637 39053306

[22] Shahsavari S Naderi M. Reliability and agreement of different spine fracture classification systems: methodologic issue. World Neurosurg. (2018) 118:383. 10.1016/j.wneu.2018.05.098 30248811

[23] Abedeen I Rahman MA Prottyasha FZ Ahmed T Chowdhury TM Shatabda S, Fracatlas: a dataset for fracture classification, localization and segmentation of musculoskeletal radiographs, Sci Data. 10 (2023) 521. 10.1038/s41597-023-02432-4 PMC10404222 37543626

[24] Bousson V Benoist N Guetat P Attané G Salvat C Perronne L. Application of artificial intelligence to imaging interpretations in the musculoskeletal area: where are we? Where are we going? Joint Bone Spine. (2023) 90:105493. 10.1016/j.jbspin.2022.105493 36423783

[25] Baur D Kroboth K Heyde CE Voelker A. Convolutional neural networks in spinal magnetic resonance imaging: a systematic review. World Neurosurg. (2022) 166:60–70. 10.1016/j.wneu.2022.07.041 35863650

[26] Lessmann N Van Ginneken B De Jong PA Išgum I. Iterative fully convolutional neural networks for automatic vertebra segmentation and identification. Med Image Anal. (2019) 53:142–55. 10.1016/j.media.2019.02.005 30771712

[27] Radke KL Müller-Lutz A Abrar DB Vach M Rubbert C Latz D. Precision through detail: radiomics and windowing techniques as key for detecting Dens axis fractures in CT scans. Diagnostics (Basel). (2025) 15:2599. 10.3390/diagnostics15202599 41153271 PMC12564139

[28] Xu F Xiong Y Ye G Liang Y Guo W Deng Q. Deep learning-based artificial intelligence model for classification of vertebral compression fractures: a multicenter diagnostic study. Front Endocrinol (Lausanne). (2023) 14:1025749. 10.3389/fendo.2023.1025749 37033240 PMC10073698

[29] Saeed MU Bin W Sheng J Albarakati HM. An automated multi-scale feature fusion network for spine fracture segmentation using computed tomography images. J Imaging Inform Med. (2024) 37:2216–26. 10.1007/s10278-024-01091-0 38622384 PMC11522210

[30] Jones MA Faiz R Qiu Y Zheng B. Improving mammography lesion classification by optimal fusion of handcrafted and deep transfer learning features. Phys Med Biol. (2022) 67:054001. 10.1088/1361-6560/ac5297 PMC8935657 35130517

[31] Liu W Zhang X Yu C Chen D Zhao K Liang J. Development and validation of a radiomics model using plain radiographs to predict spine fractures with posterior wall injury. Eur Spine J. (2025) 34:3809–19. 10.1007/s00586-025-08948-0 40410361

[32] Liu K Ning J Qin S Xu J Hao D Lang N. Identifying primary sites of spinal metastases: expert-derived features vs. ResNet50 model using nonenhanced MRI. J Magn Reson Imaging. (2025) 62:176–86. 10.1002/jmri.29720 39868626

[33] Vescio A Testa G Sapienza M Familiari F Mercurio M Gasparini G. Artificial intelligence in pediatric orthopedics: a comprehensive review. Medicina (Kaunas). (2025) 61:954. 10.3390/medicina61060954 40572641 PMC12195277

[34] Koike Y Yui M Nakamura S Yoshida A Takegawa H Anetai Y. Artificial intelligence-aided lytic spinal bone metastasis classification on CT scans. Int J Comput Assist Radiol Surg. (2023) 18:1867–74. 10.1007/s11548-023-02880-8 36991276

[35] Gong B Khalvati F Ertl-Wagner BB Patlas MN. Artificial intelligence in emergency neuroradiology: current applications and perspectives. Diagn Interv Imaging. (2025) 106:135–42. 10.1016/j.diii.2024.11.002 39672753

[36] Srinivasu PN Kumari GLA Narahari SC Ahmed S Alhumam A. Exploring the impact of hyperparameter and data augmentation in YOLO V10 for accurate bone fracture detection from x-ray images. Sci Rep. (2025) 15:9828. 10.1038/s41598-025-93505-4 40119100 PMC11928462

[37] Finetti R Roncaglia B Visibelli A Spiga O Santucci A. Data augmentation and synthetic data generation in rare disease research: a scoping review. Med Sci (Basel). (2025) 13:260. 10.3390/medsci13040260 41283261 PMC12641889

[38] Nadeem SA Comellas AP Regan EA Hoffman EA Saha PK. Chest CT-based automated vertebral fracture assessment using artificial intelligence and morphologic features. Med Phys. (2024) 51:4201–18. 10.1002/mp.17072 38721977 PMC11661457

[39] Ji C Rong Y Wang J Yu S Yin G Fan J. Risk factors for refracture following primary osteoporotic vertebral compression fractures. Pain Physician. (2021) 24:E335–40. 10.1016/j.bas.2021.100253 33988955

[40] Pereira RFB Helito PVP Leão RV Rodrigues MB Correa MFP Rodrigues FV. Accuracy of an artificial intelligence algorithm for detecting moderate-to-severe vertebral compression fractures on abdominal and thoracic computed tomography scans. Radiol Bras. (2024) 57:e20230102. 10.1590/0100-3984.2023.0102 38993956 PMC11235064

[41] Maki S Furuya T Inoue M Shiga Y Inage K Eguchi Y. Machine learning and deep learning in spinal injury: a narrative review of algorithms in diagnosis and prognosis. J Clin Med. (2024) 13:705. 10.3390/jcm13030705 38337399 PMC10856760

[42] Chen J Liu S Li Y Zhang Z Liao N Shi H. Deep learning model for automated detection of fresh and old vertebral fractures on thoracolumbar CT. Eur Spine J. (2025) 34:1177–86. 10.1007/s00586-024-08623-w 39708132

[43] Chanplakorn P Lertudomphonwanit T Daraphongsataporn N Sritara C Jaovisidha S Sa-Ngasoongsong P. Development of prediction model for osteoporotic vertebral compression fracture screening without using clinical risk factors, compared with FRAX and other previous models. Arch Osteoporos. (2021) 16:84. 10.1007/s11657-021-00957-y 34085107 PMC8175310

[44] Hassankhani A Freeman CW Banks J Parsons MS Wessell DE Hutchins TA. ACR appropriateness criteria® acute spinal trauma: 2024 update. J Am Coll Radiol. (2025) 22:S48–66. 10.1016/j.jacr.2025.02.013 40409895

[45] Liawrungrueang W Cholamjiak W Promsri A Jitpakdee K Sunpaweravong S Kotheeranurak V. Artificial intelligence for cervical spine fracture detection: a systematic review of diagnostic performance and clinical potential. Global Spine J. (2025) 15:2547–58. 10.1177/21925682251314379 39800538 PMC11726500

[46] Ruitenbeek HC Oei EHG Schmahl BL Bos EM Verdonschot R Visser JJ. Towards clinical implementation of an AI-algorithm for detection of cervical spine fractures on computed tomography. Eur J Radiol. (2024) 173:111375. 10.1016/j.ejrad.2024.111375 38377894

[47] Tee Y-S Huang J-F Huang Y-T Hsu C-P Chen H-W Hsieh C-H. Comparative analysis of AI support levels in clinical interpretation of traumatic pelvic radiographs. NPJ Digit Med. (2025) 8:518. 10.1038/s41746-025-01923-5 40804461 PMC12350962

[48] Ma D Wang Y Zhang X Su D Ma M Qian B. 3D U-net neural network architecture-assisted LDCT to acquire vertebral morphology parameters: a vertebral morphology comprehensive analysis in a Chinese population. Calcif Tissue Int. (2024) 115:362–72. 10.1007/s00223-024-01255-8 39017691

[49] Sharma S. Artificial intelligence for fracture diagnosis in orthopedic x-rays: current developments and future potential. Sicot J. (2023) 9:21. 10.1051/sicotj/2023018 37409882 PMC10324466

[50] Confavreux CB Follet H Mitton D Pialat JB Clézardin P. Fracture risk evaluation of bone metastases: a burning issue. Cancers (Basel). (2021) 13:5711. 10.3390/cancers13225711 34830865 PMC8616502

[51] Milicevic F Mahmoud MA Ghandour M Khasawneh M’Y Ghasemi AR Kabir K. Artificial intelligence in spinal surgery: current applications and future perspectives. Orthopadie (Heidelb). (2026) 55:99–104. 10.1007/s00132-025-04765-x 41563474

[52] Wagner DT Tilmans L Peng K Niedermeier M Rohl M Ryan S. Artificial intelligence in neuroradiology: a review of current topics and competition challenges. Diagnostics (Basel). (2023) 13:2670. 10.3390/diagnostics13162670 37627929 PMC10453240

[53] Sun X Jing P Yang Y Sun H Tang W Mi J. Risk prediction of osteoporotic vertebral compression fractures in postmenopausal osteoporotic women by machine learning modelling. Front Med (Lausanne). (2025) 12:1664219. 10.3389/fmed.2025.1664219 41020247 PMC12464034

[54] Feng ST Yang Y Li X Zuo WY Sun HB. Risk factors of new symptomatic fractures after vertebroplasty: a retrospective cohort study of 268 patients with painful osteoporotic vertebral compression fracture. World Neurosurg. (2024) 187:e890–7. 10.1016/j.wneu.2024.05.007 38734168

[55] Zhang ZL Yang JS Hao DJ Liu TJ Jing QM. Risk factors for new vertebral fracture after percutaneous vertebroplasty for osteoporotic vertebral compression fractures. Clin Interv Aging. (2021) 16:1193–200. 10.2147/cia.S312623 34188462 PMC8235945

[56] Zhu Z Li Y Chen J Su S Tao R Wang D. Risk factors for osteoporotic vertebral compression fracture and evaluation of clinical outcomes of minimally invasive vertebral augmentation. Global Spine J. (2026):21925682261418701. 10.1177/21925682261418701 41576219 PMC12830352

[57] Luigi-Martínez HE Layuno-Matos JG Fernández-Vélez NA Fernández-Soltero R Señeriz-Ortiz R. Artificial intelligence and augmented reality in orthopedic surgery: a narrative review of current applications and future directions. Cureus. (2025) 17:e100177. 10.7759/cureus.100177 PMC12834082 41602239

[58] Kok J Shcherbakova YM Schlösser TPC Seevinck PR Van Der Velden TA Castelein RM. Automatic generation of subject-specific finite element models of the spine from magnetic resonance images. Front Bioeng Biotechnol. (2023) 11:1244291. 10.3389/fbioe.2023.1244291 37731762 PMC10508183

[59] Burström G Buerger C Hoppenbrouwers J Nachabe R Lorenz C Babic D. Machine learning for automated 3-dimensional segmentation of the spine and suggested placement of pedicle screws based on intraoperative cone-beam computer tomography. J Neurosurg Spine. (2019) 31:147–54. 10.3171/2018.12.Spine181397 30901757

[60] Wang B Zou C Liu X Liu D Zhang Y Zang L. Development and validation of deep learning preoperative planning software for automatic lumbosacral screw selection using computed tomography. Bioengineering (Basel). (2024) 11:1094. 10.3390/bioengineering11111094 39593754 PMC11592283

[61] Colamaria A Carbone F Leone A Palmieri G Iodice S Baldassarre BM. Cervical lateral mass and pedicle fracture reduced with a herbert screw: a technical note. Med Sci (Basel). (2025) 13:92. 10.3390/medsci13030092 40700121 PMC12286195

[62] Kaya I Cingöz İD Şahin MC Atar M Ozyoruk S Sayin M. Are 3D printing templates an advantage in upper thoracic pedicle screw fixation? Cureus. (2021) 13:e13989. 10.7759/cureus.13989 33758726 PMC7978149

[63] Kim KH Lee EC Yoon YD Shin DW Koo HW Lee BJ. Translation of computed tomography images to T2-weighted magnetic resonance images of lumbar spine using generative adversarial networks on sagittal images. Sci Rep. (2025) 15:18385. 10.1038/s41598-025-03516-4 40419684 PMC12106705

[64] Pang AS Lim FNJ Sundaram PPM Oh JY. Accuracy of thoracolumbar pedicle screws using routine O-arm intraoperative navigation - A 15-year review with over 5000 screws. A new benchmark? Spine (Phila Pa 1976). (2025) 51:E169–74. 10.1097/brs.0000000000005375 41217367

[65] Lin M-S Huang C-W Tsou H-K Tzeng C-Y Kao T-H Lin R-H. Advances in surgical treatment for atlantoaxial instability focusing on rheumatoid arthritis: analysis of a series of 67 patients. Int J Rheum Dis. (2023) 26:1996–2006. 10.1111/1756-185x.14855 37565304

[66] Bourret S Cloche T Boue L Thompson W Dubois T Le Huec JC. Computed tomography intraoperative navigation in spinal surgery: assessment of patient radiation exposure in current practices. Int J Spine Surg. (2022) 16:909–15. 10.14444/8319 36153041 PMC9926940

[67] Azhar S Syafiq S Arif NK Mohamed Khalil AF Muhamad Ariffin MH. Navigated percutaneous sacroiliac screw fixation in unstable pelvic ring fracture. Cureus. (2022) 14:e29897. 10.7759/cureus.29897 36348881 PMC9631861

[68] Barsa P Fröhlich R Adamík J Suchomel P. Surgical treatment of cervical spine fractures in ankylosing spondylitis patients: posterior stabilization using intraoperative CT scanner-based navigation. Rozhl Chir. (2020) 99:212–8. 10.33699/pis.2020.99.5.212-218 32545972

[69] Franceschini C Ahmadi M Zhang X Wu K Lin M Weston R. Revolutionizing spine surgery with emerging AI-FEA integration. J Robot Surg. (2025) 19:615. 10.1007/s11701-025-02772-w 40965805 PMC12446114

[70] Ghosh A Freda PJ Shahrestani S Boyke AE Orlenko A Choi H. Preoperative anemia is an unsuspecting driver of machine learning prediction of adverse outcomes after lumbar spinal fusion. Spine J. (2025) 25:1596–607. 10.1016/j.spinee.2025.01.031 39892713

[71] Lin RT Ramanathan R Dalton JF Como CJ Lee I Gonzalez C. Prior year hospital admission predicts 30-day hospital readmission after spine surgery. N Am Spine Soc J. (2025) 23:100750. 10.1016/j.xnsj.2025.100750 40688351 PMC12272111

[72] Ma Y-H Tian Z-S Liu H-C Zhang B-Y Zhu Y-H Meng C-Y. Predictive risk factors for recollapse of cemented vertebrae after percutaneous vertebroplasty: a meta-analysis. World J Clin Cases. (2021) 9:2778–90. 10.12998/wjcc.v9.i12.2778 33969060 PMC8058688

[73] Gao X Du J Gao L Hao D Hui H He B. Risk factors for bone cement displacement after percutaneous vertebral augmentation for osteoporotic vertebral compression fractures. Front Surg. (2022) 9:947212. 10.3389/fsurg.2022.947212 35965863 PMC9366098

[74] Lee DG Bae JH. Fatty infiltration of the multifidus muscle independently increases osteoporotic vertebral compression fracture risk. BMC Musculoskelet Disord. (2023) 24:508. 10.1186/s12891-023-06640-2 37349814 PMC10286493

[75] Namireddy SR Gill SS Peerbhai A Kamath AG Ramsay DSC Ponniah HS. Artificial intelligence in risk prediction and diagnosis of vertebral fractures. Sci Rep. (2024) 14:30560. 10.1038/s41598-024-75628-2 39702597 PMC11659610

[76] Yamauchi I Nakashima H Ito S Segi N Ouchida J Morita Y. Preoperative low lumbar hounsfield units and global alignment predict postoperative mechanical complications after adult spinal deformity surgery: a multicenter retrospective study. J Clin Med. (2025) 14:4267. 10.3390/jcm14124267 40566011 PMC12194083

[77] Jin C He L Chen X Zheng J He W Han W. Risk factors for progressive kyphosis after percutaneous kyphoplasty in osteoporotic vertebral compression fracture. Open Med (Wars). (2024) 19:20241107. 10.1515/med-2024-1107 39669375 PMC11635761

[78] Zhao YL Li B Qian L Chen XM Wang YH Cui LB. Evaluation and analysis of risk factors for fractured vertebral recompression post-percutaneous kyphoplasty: a retrospective cohort study based on logistic regression analysis. BMC Musculoskelet Disord. (2025) 26:731. 10.1186/s12891-025-08979-0 40731342 PMC12305885

[79] Yang K Zhu X Sun X Shi H Sun L Ding H. Bone cement distribution patterns in vertebral augmentation for osteoporotic vertebral compression fractures: a systematic review. J Orthop Surg Res. (2025) 20:568. 10.1186/s13018-025-05868-z 40468349 PMC12135570

[80] Cai S Liu W Cai X Xu C Hu Z Quan X. Predicting osteoporotic fractures post-vertebroplasty: a machine learning approach with a web-based calculator. BMC Surg. (2024) 24:142. 10.1186/s12893-024-02427-x 38724895 PMC11080251

[81] Zhang Y Zhang Y You X Qiu X Tang W Zhang Y. Using artificial intelligence to predict residual distal lumbosacral pain post percutaneous kyphoplasty for osteoporotic vertebral compression fractures. Pain Physician. (2025) 28:E337–46. 40773631

[82] Ge C Li C Zhu Y Yang C Xu X. Comparison of artificial intelligence models using CT radiomics for predicting post-vertebral augmentation residual back pain in osteoporotic vertebral compression fractures. Int J Med Sci. (2025) 22:3329–41. 10.7150/ijms.114419 40765571 PMC12320780

[83] Wang H Qin H Yang K Ma Y Zhang W Li K. The dural sac spiking sign: predictive significance of magnetic resonance imaging-detected dural morphological abnormalities for dural tears in lumbar burst fractures. Eur Spine J. (2025) 35:1931–42. 10.1007/s00586-025-08964-0 40478252

[84] Mohamad NH Salim AA Yusof MI Khoh PS Lim HS Bahrin Z. Prevalence, implications, and risk factors of traumatic dural tears in thoracic and lumbar fractures: a retrospective study. Cureus. (2024) 16:e64351. 10.7759/cureus.64351 39131014 PMC11316621

[85] Chang C Law H Poon C Yen S Lall K Jamshidi A. Segment anything model (SAM) and medical SAM (MedSAM) for lumbar spine MRI. Sensors (Basel). (2025) 25:3596. 10.3390/s25123596 40573483 PMC12196577

[86] Ryu JS Kang H Chu Y Yang S. Vision-language foundation models for medical imaging: a review of current practices and innovations. Biomed Eng Lett. (2025) 15:809–30. 10.1007/s13534-025-00484-6 40917147 PMC12411343

[87] Paschali M Chen Z Blankemeier L Varma M Youssef A Bluethgen C. Foundation models in radiology: what, how, why, and why not. Radiology. (2025) 314:e240597. 10.1148/radiol.240597 39903075 PMC11868850

[88] Alhaug OK Furunes H Kaur S Blomfeldt N Dolatowski FC. Reliability of surgeon-reported MRI findings to a national spine register. Acta Neurochir (Wien). (2025) 167:105. 10.1007/s00701-025-06524-5 40227524 PMC11996961

[89] Khanal B Hasan SMK Khanal B Linte CA. Investigating the impact of class-dependent label noise in medical image classification. Proc SPIE Int Soc Opt Eng. (2023) 12464:128. 10.1117/12.2654420 PMC10134892 37123015

[90] Hearn J Van den Eynde J Chinni B Cedars A Gottlieb Sen D Kutty S. Data quality degradation on prediction models generated from continuous activity and heart rate monitoring: exploratory analysis using simulation. JMIR Cardio. (2023) 7:e40524. 10.2196/40524 37133921 PMC10193221

[91] Luo E Hao M Wei L Zhang X. Scdiffusion: conditional generation of high-quality single-cell data using diffusion model. Bioinformatics. (2024) 40. 10.1093/bioinformatics/btae518 PMC11368386 39171840

[92] Ding R He S Wu X Zhong L Chen G Gu R. Efficient generation of HPLC and FTIR data for quality assessment using time series generation model: a case study on Tibetan medicine shilajit. Front Pharmacol. (2024) 15:1503508. 10.3389/fphar.2024.1503508 39624838 PMC11608951

[93] El Kojok Z Al Khansa H Trad F Chehab A. Augmenting a spine CT scans dataset using VAEs, GANs, and transfer learning for improved detection of vertebral compression fractures. Comput Biol Med. (2025) 184:109446. 10.1016/j.compbiomed.2024.109446 39550911

[94] Jhaveri A Abolhassani F Fine B. Prospective external validation of an AI-based emergency department pneumonia disposition prediction tool. Can Assoc Radiol J. (2025) 76:664–73. 10.1177/08465371251320938 40008968

[95] Abbas Q Jeong W Lee SW. Explainable AI in clinical decision support systems: a meta-analysis of methods, applications, and usability challenges. Healthcare (Basel). (2025) 13:2154. 10.3390/healthcare13172154 40941506 PMC12427955

[96] Ahmed F Naz NS Khan S Rehman AU Ismael WM Khan MA. Explainable artificial intelligence (XAI) in medical imaging: a systematic review of techniques, applications, and challenges. BMC Med Imaging. (2026) 26:37. 10.1186/s12880-025-02118-w 41491470 PMC12809972

[97] Li Y Sun Q Akman A Schuller BW. Explainable AI for healthcare. Stud Health Technol Inform. (2025) 330:632–52. 10.3233/shti251454 41728714

[98] Behzad S Eibschutz L Lu MY Gholamrezanezhad A. Optimizing the power of AI for fracture detection: from blind spots to breakthroughs. Skeletal Radiol. (2025) 54:2007–24. 10.1007/s00256-025-04951-0 40407826

[99] Pe S Famiglini L Gallazzi E Bortolotto C Carone L Cisarri A. Alternative strategies to generate class activation maps supporting AI-based advice in vertebral fracture detection in x-ray images. Methods Inf Med. (2024) 63:122–36. 10.1055/a-2562-2163 40460879 PMC12133327

[100] Haupt M Maurer MH Thomas RP. Explainable artificial intelligence in radiological cardiovascular imaging-A systematic review. Diagnostics (Basel). (2025) 15:1399. 10.3390/diagnostics15111399 40506971 PMC12155260

[101] Pellisé F Haddad S Núñez-Pereira S Yilgor C Pupak A Ramírez-Valencia M. Radiomics-powered radiographic image analysis for enhanced mechanical complications prediction and surgical planning in adult spine deformity. Spine J. (2026) 26:578–85. 10.1016/j.spinee.2025.10.011 41075989

[102] Li X Zhou Y Dvornek NC Gu Y Ventola P Duncan JS. Efficient shapley explanation for features importance estimation under uncertainty. Med Image Comput Comput Assist Interv. (2020) 12261:792–801. 10.1007/978-3-030-59710-8_77 34308439 PMC8299327

[103] Zhang Q Chuang C Zhang S Zhao Z Wang K Xu J. HealthiVert-GAN: a novel framework of Pseudo-healthy vertebral image synthesis for interpretable compression fracture grading. IEEE J Biomed Health Inform. (2025) 29:7299–311. 10.1109/jbhi.2025.3572458 40402696

[104] Eltawil FA Atalla M Boulos E Amirabadi A Tyrrell PN. Analyzing barriers and enablers for the acceptance of artificial intelligence innovations into radiology practice: a scoping review. Tomography. (2023) 9:1443–55. 10.3390/tomography9040115 37624108 PMC10459931

[105] Jesus R Silva LB Pose MG Costa C. A plugin-based architecture for integrating AI services in an open-source PACS. J Imaging Inform Med. (2026). 10.1007/s10278-026-01856-9 41714575

[106] Harmon SA Tetreault J Esengur OT Qin M Yilmaz EC Chang V. Research-based clinical deployment of artificial intelligence algorithm for prostate MRI. Abdom Radiol (NY). (2025) 50:5893–902. 10.1007/s00261-025-05014-7 40418374 PMC12602619

[107] Mwigereri DG Kamotho NT Waljee AK Rego RT Weinheimer-Haus EM Alarakhiya F. Synthetic data generation of health and demographic surveillance systems data: a case study in a low- and middle-income country. JAMIA Open. (2025) 8:ooaf137. 10.1093/jamiaopen/ooaf137 41267857 PMC12628187

[108] Nasir M Siddiqui K Ahmed S. Ethical-legal implications of AI-powered healthcare in critical perspective. Front Artif Intell. (2025) 8:1619463. 10.3389/frai.2025.1619463 40673212 PMC12263600

[109] Deman F Palmieri S Broeckx G Den Berghe TV Zlobec I Schirmacher P. Practical consequences of the European union-AI act for anatomic pathology laboratories a European society of pathology and European society of digital and integrative pathology commissioned expert opinion paper. Virchows Arch. (2025) 488:789–99. 10.1007/s00428-025-04291-3 41102487 PMC13053491

[110] van Den Wittenboer GJ Van Der Kolk BYM Nijholt IM Langius-Wiffen E Van Dijk RA Van Hasselt BAAM. Diagnostic accuracy of an artificial intelligence algorithm versus radiologists for fracture detection on cervical spine CT. Eur Radiol. (2024) 34:5041–8. 10.1007/s00330-023-10559-6 38206401

[111] Silberstein J Wee C Gupta A Seymour H Ghotra SS Sá dos Reis C. Artificial intelligence-assisted detection of osteoporotic vertebral fractures on lateral chest radiographs in post-menopausal women. J Clin Med. (2023) 12:7730. 10.3390/jcm12247730 38137799 PMC10743975

[112] Kim KY Kim JH Shin J Kim MS Park S-H Chang JH. Cost-effectiveness of chest radiography using artificial intelligence for lung cancer screening in South Korea. Sci Rep. (2025) 15:45604. 10.1038/s41598-025-29600-3 41315843 PMC12753820

[113] Wulandari PI Gandomkar Z Rickard M Hooshmand S Suleiman M Brennan P. Diagnostic performance of FDA-cleared and CE-marked AI systems for mammography screening: a systematic review and meta-analysis. Eur J Radiol. (2026) 204:113136. 10.1016/j.ejrad.2026.113136 42561891

[114] Korfiatis P Kline TL Meyer HM Khalid S Leiner T Loufek BT. Implementing artificial intelligence algorithms in the radiology workflow: challenges and considerations. Mayo Clin Proc Digit Health. (2025) 3:100188. 10.1016/j.mcpdig.2024.100188 40207002 PMC11975811

[115] Chou P-H Jou TH-T Wu H-TH Yao Y-C Lin H-H Chang M-C. Ground truth generalizability affects performance of the artificial intelligence model in automated vertebral fracture detection on plain lateral radiographs of the spine. Spine J. (2022) 22:511–23. 10.1016/j.spinee.2021.10.020 34737066

[116] Weng MY Lane NE. Medication-induced osteoporosis. Curr Osteoporos Rep. (2007) 5:139–45. 10.1007/s11914-007-0008-y 18430387

[117] Aleraij S Alhowti S Ferwana M Abdulmajeed I Mutawwam IM. Effect of proton pump inhibitors on bone mineral density: a systematic review and meta-analysis of observational studies. Bone Rep. (2020) 13:100732. 10.1016/j.bonr.2020.100732 33299906 PMC7701953

[118] Chou Y-Y Wang M-S Lin C-F Lee Y-S Lee P-H Huang S-M. The application of machine learning for identifying frailty in older patients during hospital admission. BMC Med Inform Decis Mak. (2024) 24:270. 10.1186/s12911-024-02684-z 39334179 PMC11430101

[119] Yang H Chang J He W Wee CF Yit JST Feng M. Frailty modeling using machine learning methodologies: a systematic review with discussions on outstanding questions. IEEE J Biomed Health Inform. (2025) 29:631–42. 10.1109/jbhi.2024.3430226 39024091

[120] Moskven E Charest-Morin R Flexman AM Street JT. The measurements of frailty and their possible application to spinal conditions: a systematic review. Spine J. (2022) 22:1451–71. 10.1016/j.spinee.2022.03.014 35385787

[121] Mayo AM Wallhagen MI. Considerations of informed consent and decision-making competence in older adults with cognitive impairment. Res Gerontol Nurs. (2009) 2:103–11. 10.3928/19404921-20090401-08 20077971

[122] Fields LM Calvert JD. Informed consent procedures with cognitively impaired patients: a review of ethics and best practices. Psychiatry Clin Neurosci. (2015) 69:462–71. 10.1111/pcn.12289 25756740

[123] Park HJ. Patient perspectives on informed consent for medical AI: a web-based experiment. Digit Health. (2024) 10:20552076241247938. 10.1177/20552076241247938 38698829 PMC11064747

[124] Shen L Gao C Hu S Kang D Zhang Z Xia D. Using artificial intelligence to diagnose osteoporotic vertebral fractures on plain radiographs. J Bone Miner Res. (2023) 38:1278–87. 10.1002/jbmr.4879 37449775

[125] Li Y Liang Z Li Y Cao Y Zhang H Dong B. Machine learning value in the diagnosis of vertebral fractures: a systematic review and meta-analysis. Eur J Radiol. (2024) 181:111714. 10.1016/j.ejrad.2024.111714 39241305

[126] Qiu Q Huang J Yang Y Zhao Y Zhu X Peng J. Automatic AI tool for opportunistic screening of vertebral compression fractures on chest frontal radiographs: a multicenter study. Bone. (2025) 191:117330. 10.1016/j.bone.2024.117330 39549901

[127] Kumar R Marla K Sporn K Paladugu P Khanna A Gowda C. Emerging diagnostic approaches for musculoskeletal disorders: advances in imaging, biomarkers, and clinical assessment. Diagnostics (Basel). (2025) 15:1648. 10.3390/diagnostics15131648 40647646 PMC12248478

[128] Goldberg AL Kershah SM. Advances in imaging of vertebral and spinal cord injury. J Spinal Cord Med. (2010) 33:105–16. 10.1080/10790268.2010.11689685 20486529 PMC2869279

[129] Wu YM Tang FY Qi ZX. Multimodal artificial intelligence technology in the precision diagnosis and treatment of gastroenterology and hepatology: innovative applications and challenges. World J Gastroenterol. (2025) 31:109802. 10.3748/wjg.v31.i38.109802 41112005 PMC12531745

[130] Sekuboyina A Husseini ME Bayat A Löffler M Liebl H Li H. Verse: a vertebrae labelling and segmentation benchmark for multi-detector CT images. Med Image Anal. (2021) 73:102166. 10.1016/j.media.2021.102166 34340104

[131] Schuurmans M Saha A Alves N Vendittelli P Yakar D Sabroso-Lasa S. End-to-end prognostication in pancreatic cancer by multimodal deep learning: a retrospective, multicenter study. Eur Radiol. (2025) 35:7537–48. 10.1007/s00330-025-11694-y 40410330 PMC12634761

[132] Ahangari G Norioun H Ghaemi S Zali A. Artificial intelligence in glioblastoma diagnostics: integrating MRI, histopathology, and molecular profiling. Cancer Treat Res Commun. (2025) 45:101040. 10.1016/j.ctarc.2025.101040 41308410

[133] Li M Sun S Liu M Wang N Luo C Sun G. Mapping knowledge landscapes and emerging trends in AI for coronary artery disease imaging biomarkers: a bibliometric and visualization analysis. Curr Probl Cardiol. (2026) 51:103302. 10.1016/j.cpcardiol.2026.103302 41713756

[134] Schilcher J Nilsson A Andlid O Eklund A. Fusion of electronic health records and radiographic images for a multimodal deep learning prediction model of atypical femur fractures. Comput Biol Med. (2024) 168:107704. 10.1016/j.compbiomed.2023.107704 37980797

[135] Mani K Scharfenberger T Goldman SN Kleinbart E Mostafa E De La Garza Ramos R. Multimodal machine learning for predicting perioperative safety indicators in spinal surgery. Spine J. (2025) 25:2450–60. 10.1016/j.spinee.2025.03.021 40164437

[136] Liu K Qiu W Yang X. Exploring the growth and impact of artificial intelligence in anesthesiology: a bibliometric study from 2004 to 2024. Front Med (Lausanne). (2025) 12:1595060. 10.3389/fmed.2025.1595060 40529130 PMC12171226

[137] Peng Z Ma R Zhang Y Yan M Lu J Cheng Q. Development and evaluation of multimodal AI for diagnosis and triage of ophthalmic diseases using ChatGPT and anterior segment images: protocol for a two-stage cross-sectional study. Front Artif Intell. (2023) 6:1323924. 10.3389/frai.2023.1323924 38145231 PMC10748413

